# Supplementary material for: Evaluating the quality of remote sensing products for agricultural index insurance
Source: PLoS One. 2021 Oct 8;16(10):e0258215. doi: 10.1371/journal.pone.0258215 (PMC8500421; doi:10.1371/journal.pone.0258215)
Supplement: S1 Appendix — (PDF) [file pone.0258215.s001.pdf]

S1 Appendix: Variability of risk preferences to insurance compensation

S1 Fig 1 shows the impact of risk aversion on the certainty equivalent components that combine to create the RIB measure in Eq (**Error! Reference source not found.**). The dashed (red) line displays CE^N and the dot-dashed (green) line displays CE^P, the certainty equivalent for perfect insurance. Because the insurance is marked up over the actuarially fair price, a risk neutral agent ($\rho = 0$) would be better off without insurance. Perfect insurance offers positive benefits for all risk aversion levels beyond about 0.4. The index insurance contract generates certainty equivalent levels shown by the solid (blue) curve. It offers no insurance benefit at risk aversion levels below 0.6. At the moderate risk aversion level of 1.5 used in this paper's RIB calculations, we see that the index provides roughly half the gain of the perfect contract as the solid line lies mid-way between CE^P and CE^N . At every higher level of risk aversion, the RIB for the index contract declines slightly, reflecting the fact that even the best index contract occasionally fails to correctly compensate severe losses.

S1 Fig 1: Sensitivity of risk aversion to the value of an insurance contract evaluated over a range of 0-3.

S1 Table 1: RIB for the 24 combination of four regression modelling approaches: linear (lm), piecewise linear with z-scores less than 0 (lm0), piecewise linear with z-scores less than -0.5 (lm5), and segmented regression (sm), using z-scores derived from six data sources: Log MODIS NDVI (LMD), log NOAA NDVI (LNO), log rainfall (LRN), MODIS NDVI (MD), NOAA NDVI (NO), and rainfall (RN).

	lm	lm0	lm5	Sm
lMD	0.25	0.42	0.50	0.50
lNO	0.25	0.42	0.42	0.42
lRN	0.25	0.42	0.42	0.42
MD	0.17	0.42	0.50	0.50
NO	0.17	0.33	0.42	0.42
RN	0.08	0.42	0.50	0.50

S1 Table 2: R^2 for the 24 combination of four regression modelling approaches: linear (lm), piecewise linear with z-scores less than 0 (lm0), piecewise linear with z-scores less than -0.5 (lm5), and segmented regression (sm), using z-scores derived from six data sources: Log MODIS NDVI (LMD), log NOAA NDVI (LNO), log rainfall (LRN), MODIS NDVI (MD), NOAA NDVI (NO), and rainfall (RN).

	lm	lm0	lm5	sm
lMD	0.29	0.39	0.42	0.47
lNO	0.24	0.37	0.51	0.40
lRN	0.26	0.36	0.37	0.41
MD	0.26	0.38	0.42	0.46
NO	0.19	0.33	0.46	0.38
RN	0.19	0.39	0.42	0.44
