# Supplementary material for: Evaluating the quality of remote sensing products for agricultural index insurance
Source: PLoS One. 2021 Oct 8;16(10):e0258215. doi: 10.1371/journal.pone.0258215 (PMC8500421; doi:10.1371/journal.pone.0258215)
Supplement: S2 Appendix — (PDF) [file pone.0258215.s002.pdf]

S2 Appendix: Cross-validation results

When using cross-validation instead of fitting and evaluating the models with all data, the results are similar for R^2 in the sense that the segmented regression performs best, and simple linear regression performs worst (S2 Fig 1) and the results are strongly correlated, although all the R^2 values are lower with cross-validation, as expected (S2 Fig 2, S2 Table 1). However, there is more variability, and the lm5 model performs poorer in cross-validation. RIB values had a high correlation (0.92) and the best cross-validated model was also among the best model when using all values for model fitting and evaluation.

The order of the predictor variable quality also changed. For example, the median score for MODIS and rainfall data was higher in cross-validation. Given the inflexibility of our models (only 2 to 3 parameters) and somewhat small sample size, cross-validation may not improve the estimate of error, but we show the results here, as we think they are generally important to consider, especially when using more flexible models such are often used in remote sensing (e.g. RandomForest).

S2 Fig 1: Five-fold cross-validation based assessment of the quality of an insurance index using R^2 and the Relative Insurance Benefit (*RIB*) measure for different regression models (a & b) and remote sensing predictor variables (c & d). Four regression models were used: linear (lm), piecewise linear with *z*-scores less than 0 (lm0), piecewise linear with *z*-scores less than -0.5 (lm5), and segmented regression (sm). Data sources used as predictors were: Log MODIS NDVI (LMD), log NOAA NDVI (LNO), log rainfall (LRN), MODIS NDVI (MD), NOAA NDVI (NO), and rainfall (RN).

S2 Fig 2: Internal vs five-fold cross-validated R^2 and *RIB* for the regression models used.

S2 Table 1: Internal vs five-fold cross-validated R^2 and RIB for the regression models used.

		Internal		Cross-validated	
group	model	R^2	RIB	R^2	RIB
lMD	lm	0.29	0.09	0.24	0.06
lMD	lm0	0.39	0.36	0.27	0.35
lMD	lm5	0.42	0.45	0.31	0.37
lMD	seg	0.47	0.45	0.41	0.47
lNO	lm	0.24	0.09	0.18	0.12
lNO	lm0	0.37	0.36	0.31	0.38
lNO	lm5	0.51	0.36	0.41	0.42
lNO	seg	0.40	0.45	0.33	0.37
lRN	lm	0.26	0.09	0.22	0.10
lRN	lm0	0.36	0.27	0.30	0.31
lRN	lm5	0.37	0.36	0.21	0.16
lRN	seg	0.41	0.36	0.34	0.39
MD	lm	0.26	0.00	0.22	0.02
MD	lm0	0.38	0.36	0.26	0.33
MD	lm5	0.42	0.45	0.31	0.35
MD	seg	0.46	0.45	0.39	0.44
NO	lm	0.19	0.09	0.14	0.05
NO	lm0	0.33	0.27	0.27	0.32
NO	lm5	0.46	0.36	0.38	0.37
NO	seg	0.38	0.36	0.32	0.35
RN	lm	0.19	0.00	0.14	0.00
RN	lm0	0.39	0.36	0.34	0.34
RN	lm5	0.42	0.36	0.27	0.25
RN	seg	0.44	0.45	0.36	0.41
