# Supplementary figures and images for: Evaluating the quality of remote sensing products for agricultural index insurance
Source: PLoS One. 2021 Oct 8;16(10):e0258215. doi: 10.1371/journal.pone.0258215 (PMC8500421; doi:10.1371/journal.pone.0258215)

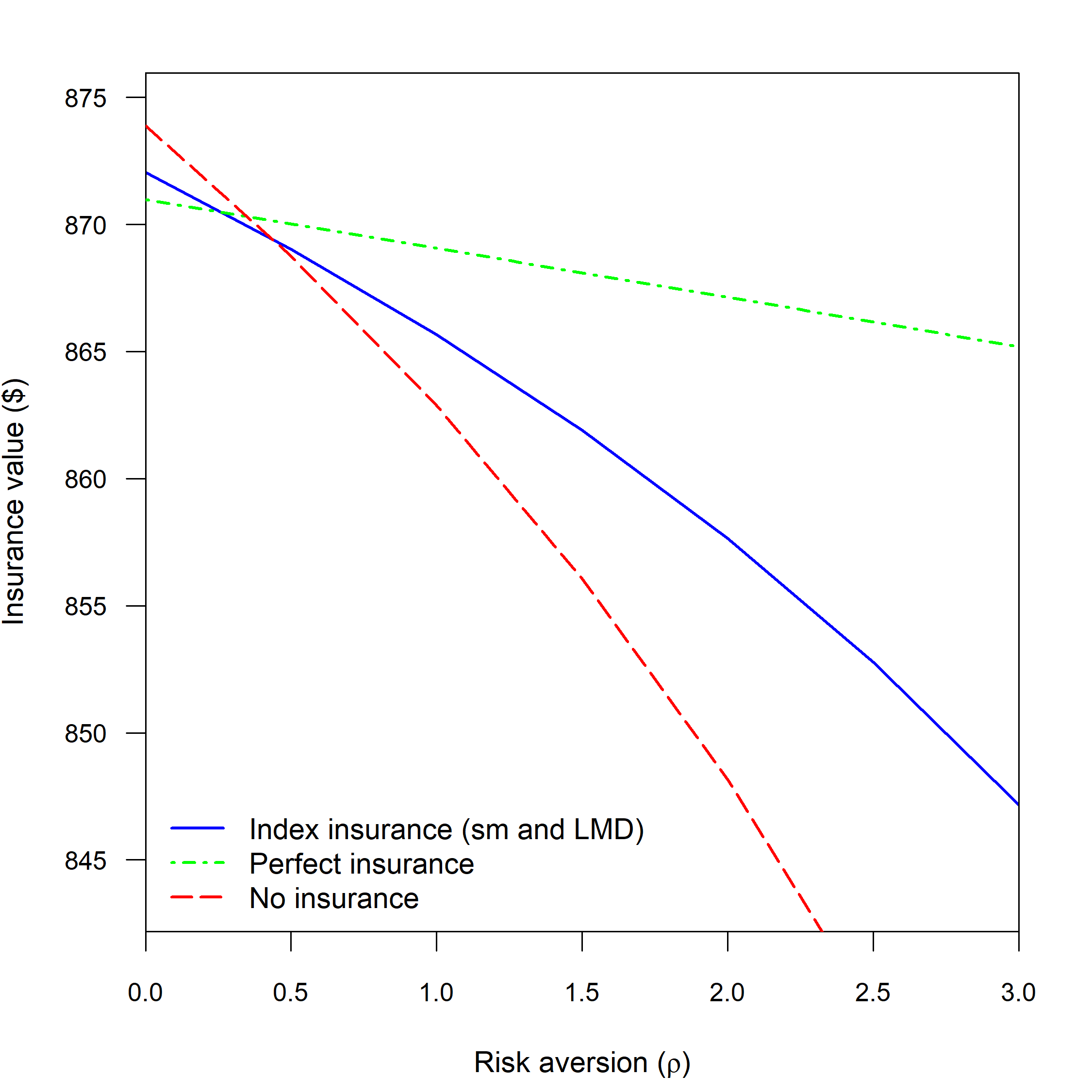

Supplement: S1 Fig — (TIF) [file pone.0258215.s003.tif]

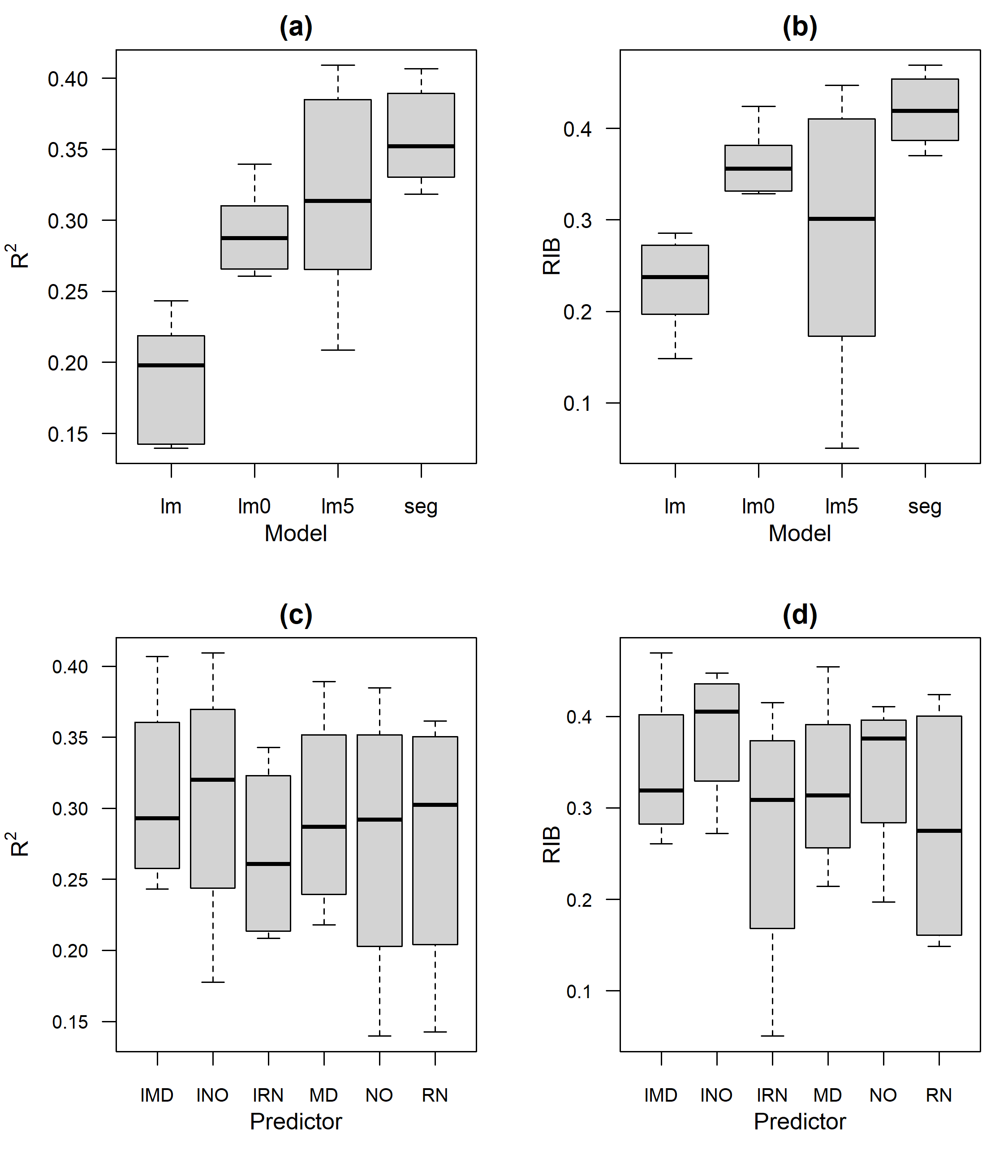

Supplement: S2 Fig — Five-fold cross-validation based assessment of the quality of an insurance index using R2 and the Relative Insurance Benefit (RIB) measure for different regression models (a & b) and remote sensing predictor variables (c & d). Four regression models were used: linear (lm), piecewise linear with z-scores less than 0 (lm0), piecewise linear with z-scores less than -0.5 (lm5), and segmented regression (sm). Data sources used as predictors were: Log MODIS NDVI (LMD), log NOAA NDVI (LNO), log rainfall (LRN), MODIS NDVI (MD), NOAA NDVI (NO), and rainfall (RN). (TIF) [file pone.0258215.s004.tif]

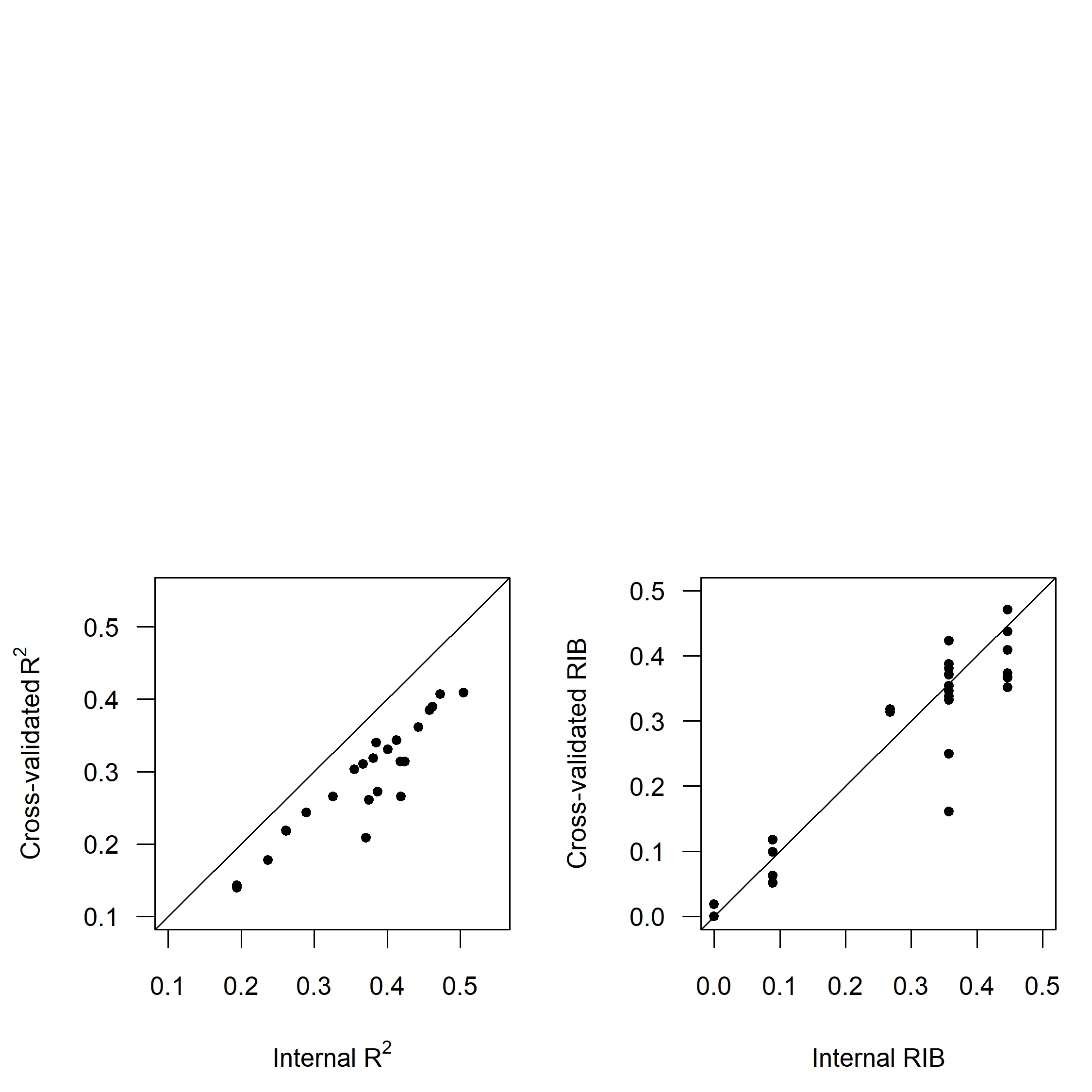

Supplement: S3 Fig — (TIF) [file pone.0258215.s005.tif]
