# Supplementary material for: Evaluating the quality of remote sensing products for agricultural index insurance
Source: PLoS One. 2021 Oct 8;16(10):e0258215. doi: 10.1371/journal.pone.0258215 (PMC8500421; doi:10.1371/journal.pone.0258215)
Supplement: Attachment — Submitted filename: PLOS one R2 Rebuttal_letter.docx [file pone.0258215.s007.docx]

# Dear Editor,

# Thank you for your suggestions including those of the reviewers to improve our manuscript titled “Evaluating the Quality of Remote Sensing Products for Agricultural Index Insurance”. We have edited the manuscript and below you find the comments (indented in italics and black font) and our responses in blue font.

Sincerely,

Benson Kenduiywo

(on-behalf of all the co-authors)

**EDITOR**

1. *Please re-consider the request to provide an out-of-sample analysis as this is clearly known to provide more robust and trustworthy model assessment than in-sample. That aside, an out-of-sample analysis could also address the concern of how data dependent the RIB is (raised by reviewer 2), irrespective of the size of the data. While i appreciate the explanation that the modelling approach is not susceptible to over-fitting, I would encourage the use of out-of-sample to clear any doubts of biased results.*

We have done the cross-validation analysis (see response to Reviewer 2).

2. *I think that the response "We used a case study on livestock insurance, but the economic framework used, and the RIB also apply to crop insurance" is a bit simplistic and requires additional details regarding the applicability in crop insurance. Yes, it should be theoretically possible to apply it to crop insurance, but as the two systems are markedly different, e.g. in terms of assessing losses, authors may provide some few lines to guide the reader or potential users on extending this to crop insurance. Else, you may decide to revise the title of the paper to "livestock index insurance" instead of "agricultural index insurance".*

We have expanded the paragraph on how our model can be adopted for the crop production case. We now state:

These same methods can be applied to crop insurance contracts. Instead of estimating livestock mortality, crop yield can be estimated, and this is a very active research area for which considerable progress in obtaining reliable estimates has been reported [1], [38]–[40]. Such remote sensing based estimates of crop yield are commonly used in index insurance [1]. Crop insurance programs can also be evaluated with expected utility-based metrics. For example, [7] measured the quality of index-based insurance for rice producers in northern Tanzania. It would be important to further evaluate remote-sensing based indices for crop insurance with the RIB measure we propose because, similar to the case of livestock mortality, the quality of low-yield estimates are more important than the quality of high-yield estimates. One way to do this would be to use ground observed yields (e.g. estimated via crop cuts [41]) and to calibrate a remote sensing-based predictor ([39], [42]). Denoting predicted yields for any given farmer $i$ at a growing season $s$ in location $l$ as  $\hat{\mu}_{\mathcal{l}s}$, we could write insurance payouts for a farmer with one unit of land as:

$$I_{ils}(k)=q\times max \left( 0, {t\times\mu}_{l}-\hat{\mu}_{\mathcal{l}s} \right)$$

(19)

where *t* is again the trigger of deductible level, $\mu_{\mathcal{l}}$ is the long-term average yield for location $\mathcal{l}$ and *q* is the unit value of the crop. Using this, or any other contract specification, we could then follow the procedures estimated above to estimate expected utility (or expected economic well-being) with and without insurance as the average of utilities computed from income using Eq (6). The insurance benefit for a farmer with a perfect contract (i.e., based on crop cut yield income estimates) and those estimated from a remotely sensed index (i.e., contract *J)* can now be estimated using Eq (11) and subsequently *RIB* using Eq (12).

**REVIEWER 1**

Reviewer #1: Big thanks to the authors for the thorough revision. That helped clarifying many things. I have two remaining issues, where I am not satisfied by the responses given.

1. *First, the authors acknowledge that similar lines of thinking (using alternative metrics cp to R2) have been already used widely in the literature on index insurance. This is standard in many ag econ journals. BUT. the big gap is that it has not been done in the remote sensing related insurance applications. I do not see the difference here. Technically, it does not matter what index I used if I talk about assessment in economic terms. Please tone down the description of your own contribution with this paper, adjust language.*

Remote sensing based indices that predict livestock mortality or crop yield have been evaluated on their own, whether it be with R^2^, RMSE or some other measure. The problem with that is that these statistics do not provide much information about how good such an index is for the purpose of using them in an insurance program (except perhaps when R^2^ is 0 or 1). This is because some errors are more important than others.

Our main contribution is to show how this problem can be solved. That is, we propose the RIB measure that compares the index to a perfect index within an economic framework that evaluates the welfare benefits of insurance.

Welfare benefits of insurance programs can obviously not be measured with model fit measures such as R^2^. We present what we think is the best available economic framework, but we acknowledge that other approaches have been used. RIB can indeed be used with any reasonable approach to estimate welfare benefits from insurance.

We clarified this in the previous round and have attempted to do so in this round by carefully editing the abstract and other parts of the paper so that we do not misrepresent what is new in our analysis and what is not.

2. *Second, I do not fully get your response on out of sample performance. It does not matter what estimator etc you use. Within sample assessment (as you do it) is biased in terms of assessing the economic performance of an insurance solution, i.e. it will be usually too optimistic if doing all in-sample. Thus, also comparisons and assessments presented here are likely biased. An out-of-sample procedure (again, widely used in ag econ journals) can address these concerns and add robustness to your conclusions. As you have sufficient data, it shall not be an issue I believe.*

We have now also done a cross-validation based evaluation that is available in the supplement (S2 Appendix) and referred to in the main text, where we say:

It is important to assure that the remote sensing models do not overfit the data, especially when using flexible model fitting algorithms. Using overfitted models would lead to overstating the quality of the index and the benefit of insurance. Cross-validation can be used to compare the difference between model fit to training and testing data, and to minimize this difference during model selection; or to compute more robust *RIB* values. As we used simple models with very few parameters, we are not especially concerned about it in our case, and there is very little scope for model simplification. A disadvantage of cross-validation is that it can create unstable estimates when working with small or modest sample sizes, as in our case. Nevertheless, we also evaluated our models using five-fold cross-validation. The goodness of fit statistics are lower (0.36 vs 0.28 on average), while the *RIB* values changed much less, on average (0.30 vs 0.27). Of the best models identified, log transformed MODIS-NDVI with segmented regression, had a slightly higher *RIB* and came out as the best model in cross-validation (see appendix S2 for details).
